# Detection of Moderate Traumatic Brain Injury from Resting-State Eye-Closed Electroencephalography

**DOI:** 10.1155/2020/8923906

**Published:** 2020-03-11

**Authors:** Chi Qin Lai, Haidi Ibrahim, Aini Ismafairus Abd. Hamid, Mohd Zaid Abdullah, Azlinda Azman, Jafri Malin Abdullah

**Affiliations:** ^1^School of Electrical and Electronic Engineering, Engineering Campus, Universiti Sains Malaysia, 14300, Nibong Tebal, Penang, Malaysia; ^2^Department of Neurosciences, School of Medical Sciences, Universiti Sains Malaysia, 16150 Kubang Kerian, Kota Bharu, Kelantan, Malaysia; ^3^School of Social Sciences, Universiti Sains Malaysia, 11800 Pulau Pinang, Malaysia; ^4^Brain and Behaviour Cluster, School of Medical Sciences, Universiti Sains Malaysia, 16150 Kubang Kerian, Kota Bharu, Kelantan, Malaysia

## Abstract

Traumatic brain injury (TBI) is one of the injuries that can bring serious consequences if medical attention has been delayed. Commonly, analysis of computed tomography (CT) or magnetic resonance imaging (MRI) is required to determine the severity of a moderate TBI patient. However, due to the rising number of TBI patients these days, employing the CT scan or MRI scan to every potential patient is not only expensive, but also time consuming. Therefore, in this paper, we investigate the possibility of using electroencephalography (EEG) with computational intelligence as an alternative approach to detect the severity of moderate TBI patients. EEG procedure is much cheaper than CT or MRI. Although EEG does not have high spatial resolutions as compared with CT and MRI, it has high temporal resolutions. The analysis and prediction of moderate TBI from EEG using conventional computational intelligence approaches are tedious as they normally involve complex preprocessing, feature extraction, or feature selection of the signal. Thus, we propose an approach that uses convolutional neural network (CNN) to automatically classify healthy subjects and moderate TBI patients. The input to this computational intelligence system is the resting-state eye-closed EEG, without undergoing preprocessing and feature selection. The EEG dataset used includes 15 healthy volunteers and 15 moderate TBI patients, which is acquired at the Hospital Universiti Sains Malaysia, Kelantan, Malaysia. The performance of the proposed method has been compared with four other existing methods. With the average classification accuracy of 72.46%, the proposed method outperforms the other four methods. This result indicates that the proposed method has the potential to be used as a preliminary screening for moderate TBI, for selection of the patients for further diagnosis and treatment planning.

## 1. Introduction

Traumatic brain injury is a trauma to the brain that is caused by a blow or jolt to the head from a blunt or penetrating object. The trauma can be caused by road traffic accident, fall, or during sports activity. In emergency situations, the well-known principle of golden hour, where the treatment should be delivered within the first 60 minutes for an out-of-hospital traumatic injury patient, could impact the medical outcome of that patient [1]. Delayed treatment can cause sequelae, such as increased intracranial pressure, edema, and cerebral dysautoregulation [2, 3]. Therefore, immediate detection is crucial for the subsequent treatment plan.

The severity of the traumatic brain injury (TBI) can be classified using a few grading scores. One of the common scores is the Glasgow coma scale (GCS) [4]. GCS classifies TBI into mild, moderate, and severe based on their eye opening response, verbal response, and motor response. The GCS score corresponding to the TBI severity is shown in Table 1. Mild, moderate, and severe TBI patients have the scores of 14-15, 9–13, and 3–8 respectively. However, moderate TBI is often difficult to be detected. Patients with moderate TBI have great variability in injury severity and acute phase course [5]. In acute phase, both intra- and intercranial injury may induce secondary brain injury that can lead to fatality [6–8]. Thus, moderate TBI detection should be done in the shortest time.

For the detection of moderate TBI, clinical imaging is useful. The golden standard for detecting moderate TBI is by using the computed tomography (CT) or magnetic resonance imaging (MRI). However, employing the CT or MRI scan to every patient is expensive and time consuming. Limited resources in the hospitals can also cause the delay of performing CT scan and MRI scan on the patient that poses the risk of moderate TBI. Furthermore, performing scans on moderate TBI patients that are in the recovery stage can disturb the sleep-wake rhythm and cause a delay in the recovery [9, 10]. In addition, repeated scans for reevaluation of TBI raise concerns over the consequences of radiations from CT scans [11].

As a potential substitution for early detection of moderate TBI for the further treatment plan, electroencephalogram (EEG) is a powerful tool [12]. Studies have suggested that biomarkers that can indicate a TBI can be found by analyzing the qEEG of frequency bands of the signal which are known as alpha, beta, theta, and gamma bands. Analysis has been done on EEG signals and it is found that there are reduction in mean alpha band frequency and an increment in theta band activity as compared to healthy people [13–16]. These findings are suggested as the biomarkers for TBI.

There are many works done on TBI detection based on EEG as it has a high temporal resolution and is able to measure brain activity directly [17]. In the work by Fisher et al. [18], EEG with the implantation of cortical somatosensory evoked electroencephalographic potentials (SSEPs) is used to detect and track, in real-time, neural electrophysiological abnormalities following head injury in an animal model. It was found that the amplitude of the signal improved over time but decreased significantly after one hour of monitoring. Their analysis found significant changes in low-frequency components and an increase of EEG entropy up to 30 minutes after the injury. From their experimental results, it is suggested that cortical SSEPs could potentially be used to rapidly detect and monitor TBI. On the other hand, McBride et al. [19] have studied the visual evoked potential EEG in TBI patients. In this study, TBI patients were required to perform memory tasks during EEG recording. Event-related Tsallis entropies were extracted as features to train a support vector machine (SVM) to discriminate between normal and TBI individuals. Their results suggested the potential of EEG as an effective method for early stage detection of TBI. A decent review is also carried out by Rapp et al. [20] on the applications of EEG on detecting TBI. From the literature, it can be seen that external stimulations are often exposed to the patients during EEG recording [21, 22]. The purpose of exposing patients to stimulations is to facilitate the diagnoses of the functionality and response of the human brain towards external stimulus [23].

However, the recording of EEG using stimulants has its limitation. Task-related paradigms rely on higher abilities of cognitive function such as attention or language comprehension [24]. Patients that suffer from moderate TBI might be in coma states in the acute phase course and they may not be able to perform a task or respond to the stimulant given. In addition, requiring patients to perform specific tasks and exposing them to stimulants will disturb their sleep-wake rhythm and affect their recovery process [9, 10]. Therefore, resting-state EEG is a better alternative. It is recorded when the patient is resting with their eyes closed, providing an advantage of not disturbing the patient's sleep cycle.

We reviewed four similar computational intelligence approaches, which are used to classify severe or mild TBI from healthy samples, respectively. In the work of den Brink et al. [24], a Naive Bayes classifier was used to classify severe TBI patient and healthy control. The classifier was trained based on features of average power from the beta band of each electrode and EEG connectivity of delta, theta, and gamma bands extracted from resting-state EEG. Their method first preprocessed the signal by applying a notch filter to remove line noise, followed by a low-pass filter at 100 Hz. Next, a high-pass filter with a 0.5 Hz cutoff is applied. The artifacts in the signal are removed manually. The resulting signals of each subject are divided into two-second segments. Subsequently, the three features were extracted from the resultant segments. The connectivity of the three bands is obtained by computing the correlation between the log-transformed orthogonalized amplitude envelopes of delta, theta, and gamma bands [24]. Their approach is able to present high classification accuracy. Nonetheless, their work approach heavily relies on the extracted features. Therefore, extensive exploration has to be done to select discriminative features to ensure an effective classifier learning.

McNerney et al. [25] make use of resting-state EEG and adaptive boosting (AdaBoost) for classification of mild TBI. First, a band-pass filter with cutoff frequency from 0.1 Hz to 100 Hz is applied. Subsequently, artifacts and spikes are manually marked and removed from the signal. The features that are extracted in their method are power spectral densities (PSD) of delta, theta, alpha, and gamma bands. The PSD are calculated for signals of channels AF7 to FpZ and AF8 to FpZ. The base 10 logarithms of the average PSD for each frequency bands are used as features to train the AdaBoost classifier. AdaBoost is a powerful classifier that creates a highly accurate classifier by combining several weak and inaccurate classifiers, creating a cascade of classification model. It carries advantages such as being simple and requiring less tweaking of parameters to achieve high classification result despite being sensitive to noisy data and outliers. Thus, preprocessing becomes an unavoidable stage in the work of McNerney et al. [25] to remove external noises.

In the work of Cao et al. [26], an automatic classification of athletes with concussion has been proposed by using an EEG-based support vector machine (SVM). Their approach is able to detect mild TBI in athletes and determine whether they are suitable to return-to-play (RTP) or not. The resting-state EEG has been recorded from the subjects in three different conditions, where the subject is seated, standing on a firm surface, and standing on a foam surface. Artifacts were removed from the recorded EEG manually, by using visual inspection. One minute of artifact-free EEG is then band-pass filtered between 0.5 and 30 Hz with zero phase shift. A fast Fourier Transform (FFT) is performed on the resultant signal and the signal was divided into theta, alpha, beta1, beta2, and beta3. Power averages were calculated for each of the frequency bands. In addition to the feature set, power averages for individual 1 Hz frequency between 1 and 30 Hz for all the electrodes were computed. In order to reduce the huge feature size, feature reduction was performed using heuristic minimal redundancy maximal relevance (MRMR) framework. The features were ranked based on mutual information. Top 10 features were selected and directed to an SVM for classification of the healthy subject and mild TBI patient.

Previously, we also has proposed one method to classify moderate TBI patients and healthy subjects using the resting-state eye-closed EEG [27]. Similar to the work of Cao et al. [26], our method is also using SVM as our computational intelligence method. However, our feature to be fed to the SVM is the power value, extracted from the alpha band.

EEG recording is often contaminated with unwanted elements such as noises and artifacts. Preprocessing is crucial to remove all the unwanted elements in a signal [28]. However, it is time consuming to locate and remove the impurities in the signal. Eventually, both feature selection and preprocessing the EEG are complicated and time consuming. Furthermore, analysis of resting-state EEG can be even more challenging as it contains less information as compared with EEG with external stimulants. It is preferable for the machines to find and learn the data itself, especially the implementation of resting-state EEG.

In order to overcome the complex design of preprocessing, feature extraction, and feature selection, CNN is one of the common computational intelligence methods used in development that requires classification [29]. CNN is a machine learning method which is inspired from the biological system [30], which was originally proposed for image classification task [31]. Due to its great potential in analysis of small details presented by pixels in an image, CNN is also applicable for EEG analysis [32–34]. This is because the data points of the EEG can be arranged in matrix form, which is similar to the matrix of pixels [35].

The topology of CNN is made up of multilayer perception (MLP), combining the input layers, hidden layers, and output layers. The hidden layers include the convolutional layer and the conventional backpropagation neural network dense layer. The convolutional layers are made up of convolutional kernels that carry learnable parameters which require multiple iterations of learning and validation to determine the optimum value empirically [36]. The convolutional layers play the role of extracting important features from the input matrix through the weighted learnable kernels [37]. Each forward input of the matrix computes a feature map. The convolutional layers learn to activate the feature maps when the patterns of interest are detected in the input. Activated feature maps will be downsampled by using the pooling layer and further fed forward to the next layers. Fully connected layer (also known as dense layer) is trained using the feature map. The learning process of the learnable parameters implies backpropagation [31] and gradient descent [38].

The objective of this paper is to propose an eye-closed, resting-state EEG-based moderate TBI detection method using CNN. The proposed method can avoid human error and potentially become an early screening tool for TBI in the emergency department. The parameters of the CNN are selected empirically for an optimum tuning of the architecture. The parameters are the learning rate and the mini batch size. Our method is further compared to existing state-of-art approaches and our previous work [27].

## 2. Methods

### 2.1. Subjects

The dataset that is used in this study was collected at the Hospital Universiti Sains Malaysia, Kelantan, Malaysia. Ethical approval has been obtained from Universiti Sains Malaysia, with reference number USM/JEPeM/1511045. A total of 30 resting-state eyes-closed EEG recordings were collected from 30 subjects, which are divided into 15 moderate TBI patients and 15 healthy volunteers. The TBI data was contributed by 15 patients. The healthy data were collected from 15 healthy persons. The age range for moderate TBI subjects is between 18 to 65 years old. All of them sustained nonsurgical moderate TBI according to the GSC, corresponding to a score between 9 and 13, where all of them suffer the initial hit involving the left frontal-temporal-parietal lobe as diagnosed by CT scan of the brain. Each of the subjects is required to close their eyes during the recording to obtain the resting-state EEG data.

### 2.2. Recording System and Electrode Placement

**Figure 1:**
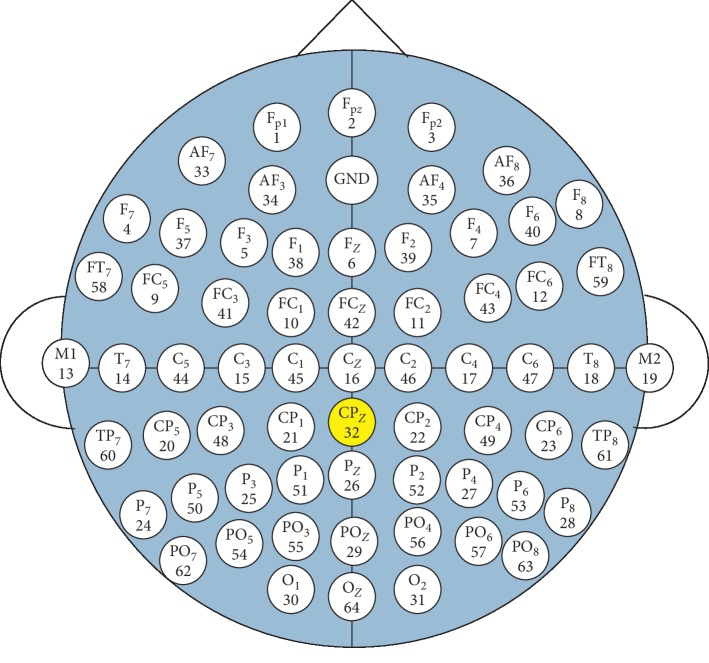
Arrangement of EEG channels on the WaveGuard EEG cap.

The EEG signals were continuously recorded by using 64 electrodes mounted on a 64-channel WaveGuard EEG cap. The placement of the channels is based on the international 10-10 EEG electrode system, which is shown in Figure 1. The electrical activities from the scalp will be recorded at 64 sites. However, *CP*_*z*_ channel recording is excluded in this study, leaving only 63 useful channels, because *CP*_*z*_ channel is used as an electrooculography (EOG) channel in this study. The ground electrode is located 10% anterior to Fz, linked earlobes served as reference and electrode impedances are below 5 kOhm. EEG signals are recorded using a programmable DC coupled broadband SynAmps amplifier. The EEG signals are amplified (gain 2500, accuracy 0.033/bit) with a recording range set for ±55 mV in the DC to 70-Hz frequency range. The EEG signals are digitized at 1000 Hz using 16-bit analog-to-digital converters.

### 2.3. Data Preparation

The first 60 seconds of the recording are discarded as they are normally contaminated by artifacts because subjects are usually not calm enough at the early phase of the recording. Segments that contained artifacts are removed based on inspection. Next 60 seconds of the recording is then divided into 60 segments of one second each. A study has shown that 60 seconds of recordings is sufficient for obtaining reliable diagnosis results [39]. In addition, the presence of more discriminative characteristic of EEG is close to the beginning of the recording [40].

As the input to the CNN, the EEG is arranged in the form of matrix of amplitude of the channel versus time. The arrangement of the channels refers to the default arrangement given by the 64-channel WaveGuard EEG cap. Because each segment is in one second, the matrix size of the EEG is *N* × *F*_*s*_, where *N* is the number of channels and *F*_*s*_ is the sampling frequency. In this research, the matrix size is 63 × 1000 because the sampling rate of 1000 Hz is used and the number of channels is 63. Therefore, each data contain 60 matrices. The components in the matrix is stored from the EEG data points using the formula:

(1) $$\begin{array}{c} M\left(i,t\right)=x_{i}\left(t\right), \end{array}$$

where *i* is the channel of the sampling point (i.e., *i*=1,2,…, *N*), *t* is the index of the sampling point (i.e., *t*=1,2,…, *F*_*s*_), and *x*_*i*_(*t*) is the amplitude of the sampling point of channel *i* at time *t*.

### 2.4. Convolutional Neural Network Topology

**Figure 2:**
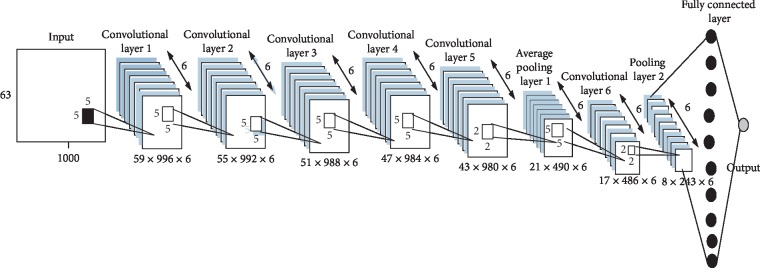
CNN topology.

The CNN topology used in this study is shown in Table 2 and Figure 2. The CNN topology used in this study is made up of six convolutional layers, two pooling layers, and one fully connected layers. Each convolution layer consists of six 5 × 5 filters. A smaller filter size is selected in order to capture finer orientation and information from the signal. Six filters are used in one convolution layer to create a feature map consisting of more variation of feature from the input. The CNN architecture is made up of nine layers in total.

The input to the CNN is a 63 × 1000 matrix. The filter size of the convolution layers used in this study is fixed to 5 × 5. The input with size *h* × *w* will generate a feature map of size *h*′ × *w*′ × *l*′ by a convolution layer, which can be calculated using

(2) $$\begin{array}{c} h^{\prime }=\left[\frac{h-f+s}{s}\right], \end{array}$$

(3) $$\begin{array}{c} w^{\prime }=\left[\frac{w-f+s}{s}\right], \end{array}$$

where *f* is the size of filter, *l*′ is the number of filter in the convolution layer, and *s* is the stride length. In this study, *f* is set as five, *l*′ is set as six, and *s* is set as one for all convolution layers.

After the first convolution layer, a feature map of 59 × 996 × 6 is produced. The feature map is next directed to the second convolution layer, outputting a feature map with size 55 × 992 × 6. After passing through the third convolution layer, a feature map of 51 × 988 × 6 is generated. Going through the fourth convolution layer, a 47 × 984 × 6 feature map is produced. Subsequently, the fifth convolution layer outputs a 43 × 980 × 6 feature map.

Next, the feature map will go through an average pooling layer. Input feature map of size *h* × *w* × *l* will generate an output feature map of size *h*′ × *w*′ × *l*′ using (2)–(4). *f* is set as two, *l*′ is set as six, and *s* is set as two for all average pooling layers in this study. The average pooling layer generated a 21 × 490 × 6 feature map. The resulting feature map will be passed to the last convolution layer, producing a 17 × 486 × 6 feature map. The feature map is then passed to an average pooling layer, which generates a 8 × 243 × 6 feature map. The output is then flattened and passed to the fully connect layer. The activation function used for the fully connected layer is Softmax. Processing of the input throughout the CNN can be visualized in Figure 2. For the CNN topology used in this study, batch normalization and rectified linear unit (ReLU) are used after each convolutional layer.

There are seven parameters that are chosen in this study for an optimum CNN topology.  Table 3 shows these parameters. The learning rate of 0.0001 is selected and remains constant throughout the training of the CNN. *L*_2_ normalization is used to perform batch normalization after every convolution layer. The mini batch size for every iteration is set as 128. As the epoch consists of 680 training data, six iterations are needed to complete one epoch passing through the CNN. The training iteration per epoch is fixed with 30. To prevent overfitting in this design, *L*_2_ regularization is used with regularization faction of 0.0005. The optimizer used for the backpropagation for CNN training is the stochastic gradient descent (SGD) with momentum of 0.9.

### 2.5. Training Procedure

In this study, the performance measure that is used to evaluate the training for the CNN is measured using classification accuracies in terms of percentage, which is testing accuracy and validation accuracy. The testing and validation accuracies are obtained using a threefold cross validation. The division of dataset for the fourfold cross validation is shown in Table 4. In this table, *k* is the number of fold, and each of the subjects is labeled as training or testing dataset on each *k*-fold validation.

The testing accuracy is calculated by using the following formula:

(4) $$\begin{array}{c} \text{Accuracy}=\frac{\text{TP}+\text{TN}}{\text{TP}+\text{TN}+\text{FP}+\text{FN}}, \end{array}$$

where TP is the moderate TBI input which is predicted correctly as moderate TBI, TN is the healthy control input that is predicted correctly as the healthy subject, FN is the TBI input that is predicted wrongly as healthy subject, and FP is the healthy control input that is predicted wrongly as moderate TBI patient. Testing accuracy is obtained using the testing set, while validation accuracy is obtained using the training set itself.

In the application of bioinformatics, small dataset often becomes an issue due to unforeseen restrictions, such as limited amount of patients. Small dataset can cause evaluation of the classifier to be optimistic biases, which is inaccurate in estimating its performance. Data augmentation can be done to increase the number of dataset, which is commonly seen in image classification. However, augmentation of moderate TBI patient's EEG can increase the classification error as random noises can be added in the process of augmentation. To overcome small dataset issue in the evaluation of the proposed architecture, bootstrap method is used in this study [41]. Bootstrap method is a resampling approach that generates bootstrap sample sets. The bootstrapping concept that generates the bootstrap sample set can be explained in three steps. First, a random sample will be selected from the original dataset. Next, the random sample will be added to the new dataset and returns to the original dataset. The two steps repeat until the bootstrap sample set reaches the fixed number of samples. For computational intelligence approach, the bootstrap sample sets that are generated will be the number of data of the original dataset [42]. Therefore, some samples will be represented repetitively, while some will not be selected at all [42]. Bootstrapping is a useful approach as the prediction results from the trained machine learning model using the bootstrap sample sets often present a Gaussian distribution. Moreover, 95% confidence interval (CI) can be calculated from the prediction results to estimate the accuracy and stability of the machine learning model.

In this study, the proposed architecture is tested by 100 iterations of resampled bootstrap sample set. It was suggested by Efron that the iterations be in the range of 50 to 200 [41]. Threefold cross validation is performed on each bootstrap sample and the cross-validation accuracy was recorded for each generated bootstrap sample set. 95% confidence interval (CI), mean cross validation accuracy (ACC), and standard deviation (SD) are calculated from the recorded cross-validation accuracies.

## 3. Results

To select the optimum learning rate and mini batch size for the training of CNN, experiments are carried out by validating the performance of different parameters. The parameters are the learning rate and mini batch size. Trained CNN models are then compared with Naive Bayes [24], AdaBoost classifier [25], SVM (MRMR) [26], and SVM (power) [27].

### 3.1. Selection of Optimum Learning Rate

Learning rate is an important parameter that determines the update step for backpropagation learning [43]. It controls the adjustment of the learnable weights with respect to the loss gradient. When the learning rate is too huge, the gradient descent can recklessly increase rather than decrease the training error. On the other hand, using learning rate which is too small can cause slow training and might cause invariable high training error. Therefore, determining the optimum learning rate is crucial, as it will affect the search of the minimum point of loss in the backpropagation learning.

**Figure 3:**
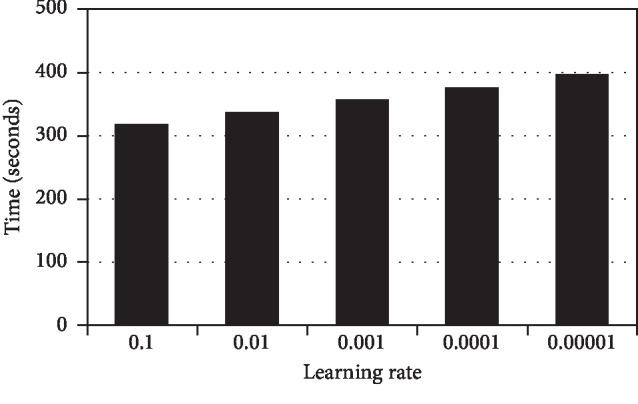
Training time by different learning rate.

The current study shows that a good learning rate can be estimated by initiating a low learning rate and increasing it at each iteration [44]. Experiments are carried out by varying the learning rate using the CNN topology with six convolutional layers and 32 mini batch size. Five learning rate values have been investigated. The learning rates used are 0.1, 0.01, 0.001, 0.0001, and 0.00001, respectively. The training times for the CNN using different learning rate are recorded and shown in Figure 3. This graph shows that a longer training time is needed for smaller value of the learning rate. Besides, from the experiment, the means of 3-fold cross-validation accuracy, SD, and 95% CI are shown in Table 5. From this table, it is shown that the learning rate of 0.0001 gives the best performance, in terms of the mean accuracy and SD.

### 3.2. Determining the Optimum Mini Batch Size

In CNN learning, training set is divided into numbers of mini batches, each consisting of a small number of training samples. The mini batch size is one of the parameters that has to be determined empirically for an optimized CNN topology. A larger mini batch can lead to a faster CNN training. However, a large mini batch uses high computational power. In addition, a study has shown that using a mini batch size that is too large can cause significant degradation in the quality of the trained CNN model [45]. Therefore, the optimum mini batch size has to be determined to ensure a better convergence rate and a better stability of the CNN training [43].

In this experiment, mini batch size of 32 has been used as the starting point based on recommendation by some studies [36, 46]. Mini batch sizes of 32, 64, and 128 were evaluated to select the optimum mini batch size using six convolution layers CNN topology and learning rate of 0.0001.  Table 6 shows their testing accuracies, respectively. From this table, it is shown that the mini batch size of 128 gives the best performance.

### 3.3. Comparison of the Proposed Method with Existing Works

The proposed method is compared with four existing methods which are similar, as thereis no existing work that classifies moderate TBI from healthy group. The first method for comparison is the work done by den Brink et al. [24] which uses task-free EEG and Naive Bayes classifier for TBI classification. The second method that is compared was proposed by McNerney et al. [25] that uses AdaBoost classifier. The third method that is compared is the work done by Cao et al. [26] that uses SVM. The fourth method that is compared is our previous work that proposed an EEG-based SVM classifier using alpha band power for moderate TBI detection [27]. For a fair comparison, the same dataset and training procedure are used. Mean, SD, and CI of cross-validation accuracy (ACC) for different approaches using the same dataset are shown in Table 7.

## 4. Discussions

From Table 5, results show that the learning rate of 0.0001 presents the highest accuracy, which is 56.57%. At this learning rate, the step is optimum to search for the best weights of the CNN, as compared to other learning rate values. By using a larger learning rate, the step taken might over-shoot and miss out the local minimal. Meanwhile, using a lower learning rate can cause a longer CNN learning time. In Figure 3, it can be seen that the training time increases when the learning rate increases.

For the selection of suitable mini batch size, it is shown in Table 6 that mini batch size of 128 gives the highest testing accuracy. Mini batch size of 32 presents the lowest accuracy (56.57%) as it converges to a flat minimal, giving a lower testing accuracy. Mini batch size of 128 can efficiently generalize the data and converge to a sharp minimal, giving the trained CNN model a higher testing accuracy of 72.46%.

Comparing to other existing methods, the proposed method reaches a high accuracy of 72.46%, which stands out compared to the work by den Brink et al. [24], McNerney et al. [25], Cao et al. [26], and our previous work [27]. By using the same dataset, these approaches achieve the mean cross-validation accuracies of 59.05%, 54.00%, 51.17%, and 49.64%, respectively, as shown in Table 7.

Having established that the features that are extracted from the frequency bands can provide important information during the training on classifier, den Brink et al. [24] and McNerney et al. [25] both performed feature extractions relying on the frequency bands. On the other hand, for the proposed method in this paper, the raw signal did not undergo any feature extraction. The EEG is arranged in matrix form and fed to the input of the CNN topology. The convolution layers perform feature extraction to obtained distinct features from the input. The convolution layers that are made up of learnable kernals aim at extracting local features from the input. The feature extraction that take place in the convolution layers started by extracting low level features and subsequently progressed to extract higher level features.

In comparison of mean cross-validation accuracies, the proposed method outperforms the other four approaches. Naive Bayes makes assumptions that each feature is independent from each other, which removes the dependency between channels of EEG. It caused the correlations between channels to be ignored which can cause information to be lost in the process of classifier training. Therefore, the proposed method that makes use of CNN can overcome the shortcoming of Naive Bayes. AdaBoost classifier is a machine learning method which requires less tweaking of parameters and is easy to use. However, it is sensitive to noises and outliers, which is unavoidable in EEG recordings. Therefore, more efforts have to be done to ensure noises and artifacts have to be totally removed to ensure an effective classifier training. The proposed method using CNN does not require filtering of the signals to discard noises. The learnable kernels of the convolutional layers can effectively extract the important features and at the same time reject noises in the signal.

In the work by Cao et al. [26], a MRMR feature selection framework was employed to reduce the size of the large feature set. However, it shows low detection accuracy when our dataset is used. In the original work of Cao et al. [26], the EEG dataset that are used require subjects to be in three different postures when the EEG is recorded. In our case, the EEG is recorded when the subjects are relaxed and seated. Therefore, the features that are extracted using their method do not provide enough information to the SVM. The multiposture EEG that is used in their work supplies more variation of information to the classifier. On the other hand, it results in a large dataset, where feature selection has to be performed. In the process of feature selection, information lost may take place and cause reduction in detection accuracy. In our proposed method, feature extraction and selection are automated by the kernals, where the learnable parameters of these kernals are updated using the backpropagation. The automated process is more efficient compared to their approach. The efficiency of feature extract using CNN can avoid tedious feature selection and reduction process, as well as human bias.

In comparison with our previous work [27], alpha band power was extracted from the EEG as features to train a SVM. However, it has shown a lower classification than our proposed method. Alpha band power can be included as one of the features for moderate TBI classification, but using alpha band power alone is not sufficient. To provide sufficient information to train a SVM, other features have to be extracted, like correlation coefficient, phase difference, and more.

## 5. Conclusion

From this study, it was shown that the number of convolution layer, learning rate, and mini batch size are important parameters that have to be determined empirically for a design of a robust CNN. Values of parameters may vary for different applications. In the application of a CNN with six convolution layers, it was found that the learning rate of 0.0001 and a mini batch size of 128 give the best classification accuracy for moderate TBI classification purpose. The proposed method is further compared with four existing TBI classification approaches. Result indicates that the proposed method outperforms the others in terms of cross-validation accuracy as well as the ease of execution. This study has suggested that CNN is a potential substitution for EEG machine learning application which required complex procedure for preprocessing of the signals and feature extraction. Further improvement of this study can potentially introduce an immediate diagnosis tool at the emergency department for moderate TBI patients which can be used as a second opinion for physicians.

## Figures and Tables

**Table 1:** TBI severity based on GCS score.

GCS score	Traumatic brain injury severity
14-15	Mild
9–13	Moderate
<9	Severe

**Table 2:** Layers of CNN and kernel size.

Index	Layer	Kernel size	Number of filter
1	Convolution layer	5 × 5	6
2	Convolution layer	5 × 5	6
3	Convolution layer	5 × 5	6
4	Convolution layer	5 × 5	6
5	Convolution layer	5 × 5	6
6	Average pooling layer	2 × 2	—
7	Convolution layer	5 × 5	6
8	Average pooling layer	2 × 2	—
9	Fully connected layer	—	—

**Table 3:** Parameters and values.

Parameter	Setting
Learning rate	0.0001
Batch normalization	*L*_2_ normalization
*L*_2_ regularization	0.0005
Mini batch size	128
Optimizer	Stochastic gradient descent
Training repetitions per epoch	30
Momentum	0.9

**Table 4:** Data division for threefold cross validation.

	*k* = 1	*k* = 2	*k* = 3
Patient 1	Training	*Testing*	Training
Patient 2	Training	*Testing*	Training
Patient 3	Training	*Testing*	Training
Patient 4	Training	*Testing*	Training
Patient 5	Training	*Testing*	Training
Patient 6	Training	Training	*Testing*
Patient 7	Training	Training	*Testing*
Patient 8	Training	Training	*Testing*
Patient 9	Training	Training	*Testing*
Patient 10	Training	Training	*Testing*
Patient 11	*Testing*	Training	Training
Patient 12	*Testing*	Training	Training
Patient 13	*Testing*	Training	Training
Patient 14	*Testing*	Training	Training
Patient 15	*Testing*	Training	Training
Healthy 1	Training	*Testing*	Training
Healthy 2	Training	*Testing*	Training
Healthy 3	Training	*Testing*	Training
Healthy 4	Training	*Testing*	Training
Healthy 5	Training	*Testing*	Training
Healthy 6	Training	Training	*Testing*
Healthy 7	Training	Training	*Testing*
Healthy 8	Training	Training	*Testing*
Healthy 9	Training	Training	*Testing*
Healthy 10	Training	Training	*Testing*
Healthy 11	*Testing*	Training	Training
Healthy 12	*Testing*	Training	Training
Healthy 13	*Testing*	Training	Training
Healthy 14	*Testing*	Training	Training
Healthy 15	*Testing*	Training	Training

**Table 5:** Mean, SD, and CI of cross-validation accuracy (ACC) for different learning rate.

Learning rate	Mean ACC	SD	95% CI
0.1	50.12	0.27	[49.93 50.31]
0.01	51.85	1.20	[50.99 52.71]
0.001	52.02	2.83	[50.00 54.04]
0.0001	56.57	9.07	[50.08 53.06]
0.00001	52.68	2.48	[50.90 54.45]

**Table 6:** Mean, SD, and CI of cross-validation accuracy (ACC) for different mini batch size.

Mini batch size	Mean ACC	SD	95% CI
32	56.57	9.07	[50.08 53.06]
64	55.04	7.13	[52.87 57.21]
128	72.46	1.90	[67.73 77.19]

**Table 7:** Mean, SD, and CI of cross-validation accuracy (ACC) for different approaches.

Approach	Mean ACC	SD	95% CI
Naive Bayes [24]	59.05	0.05	[58.95 59.15]
AdaBoost [25]	54.00	0.72	[52.58 55.42]
SVM (MRMR) [26]	49.64	5.09	[48.63 50.65]
SVM (power) [27]	51.17	0.73	[49.72 52.62]
Proposed method	72.46	1.90	[67.73 77.19]

## Data Availability

The resting-state eye-closed electroencephalography (EEG) data used to support the findings of this study are restricted by the Human Research Ethics Committee of Universiti Sains Malaysia (USM) in order to protect patients' privacy.
